# Supplementary material for: Efficacy of Remdesivir and Neutralizing Monoclonal Antibodies in Monotherapy or Combination Therapy in Reducing the Risk of Disease Progression in Elderly or Immunocompromised Hosts Hospitalized for COVID-19: A Single Center Retrospective Study
Source: Viruses. 2023 May 19;15(5):1199. doi: 10.3390/v15051199 (PMC10222024; doi:10.3390/v15051199)
Supplement: Supplementary file 1 [file viruses-15-01199-s001.zip › viruses-2400469-supplementary.pdf]

Supplementary

Table S1. Standardized differences of variables used to generate the IPTW model.

	**Standardized Differences before Applying IPTW (%) [a]**	**Standardized Differences after Applying IPTW (%) [a]**
**Variables influencing Treatment assignment**		
Age	21.4%	6.4%
Sex	16.3%	7.9%
Secondary Infections	11.5%	2.8%
Time from Symptom Onset to Therapy	3.3%	4.3%
COVID-19 Severity at admission	11.6%	4.6%

Legend: [a] Absolute value of standardized differences displayed. A standardized difference greater than 10% is considered to indicate meaningful imbalance between groups. Overidentification test for covariate balance: $\chi 2 = 4.41364$; $p = 0.6209$. IPTW = inverse probability of treatment weighting.
